# Optimization and Modeling of Process Parameters in Multi-Hole Simultaneous Drilling Using Taguchi Method and Fuzzy Logic Approach

**DOI:** 10.3390/ma13030680

**Published:** 2020-02-03

**Authors:** Muhammad Aamir, Shanshan Tu, Majid Tolouei-Rad, Khaled Giasin, Ana Vafadar

**Affiliations:** 1 School of Engineering, Edith Cowan University, Joondalup, WA 6027, Australia; m.aamir@ecu.edu.au (M.A.); m.rad@ecu.edu.au (M.T.-R.); a.vafadarshamasbi@ecu.edu.au (A.V.); 2 Faculty of Information Technology, Beijing University of Technology, Beijing 100124, China; 3 School of Mechanical and Design Engineering, University of Portsmouth, Portsmouth PO1 3DJ, UK; khaled.giasin@port.ac.uk

**Keywords:** one-shot drilling, multi-hole drilling, hole quality, optimization, Taguchi method, fuzzy logic

## Abstract

In industries such as aerospace and automotive, drilling many holes is commonly required to assemble different structures where machined holes need to comply with tight geometric tolerances. Multi-spindle drilling using a poly-drill head is an industrial hole-making approach that allows drilling several holes simultaneously. Optimizing process parameters also improves machining processes. This work focuses on the optimization of drilling parameters and two drilling processes—namely, one-shot drilling and multi-hole drilling—using the Taguchi method. Analysis of variance and regression analysis was implemented to indicate the significance of drilling parameters and their impact on the measured responses i.e., surface roughness and hole size. From the Taguchi optimization, optimal drilling parameters were found to occur at a low cutting speed and feed rate using a poly-drill head. Furthermore, a fuzzy logic approach was employed to predict the surface roughness and hole size. It was found that the fuzzy measured values were in good agreement with the experimental values; therefore, the developed models can be effectively used to predict the surface roughness and hole size in multi-hole drilling. Moreover, confirmation tests were performed to validate that the Taguchi optimized levels and fuzzy developed models effectively represent the surface roughness and hole size.

## 1. Introduction

Conventional drilling is considered to be the most economical and efficient machining processes in the aerospace and automotive industries [1]. The most important characteristics of drilled-hole quality are surface roughness (SR) and the deviation of the hole size (HS) from its nominal diameter [2]. This is because aircrafts constantly operate under vibration/shock conditions where thousands of fastener holes in the fuselage skins are prone to fatigue [2]. Fatigue cracks normally initiate and propagate after the fastened holes create regions of concentrated stress; therefore, the reliability of aircraft structures depends on their fatigue life, which is directly related to hole quality [3]. In addition, the drilling process is normally amongst the last steps of final assembly of fabricating parts, where poor hole quality might lead to part rejection, which can become costly if optimum process parameters, tools, or drilling operations are not taken into consideration [4]. 

The Taguchi method is useful for determining the best combination of factors under desired experimental conditions [5]. The Taguchi method reduces a large number of experiments that could be required in traditional experiments when the number of process parameters increases [6]. In the Taguchi method, an orthogonal array is designed that studies the entire parameter space with a small number of experiments [7]. Another statistical method is the analysis of variance (ANOVA) which is used for the interpretation of experimental data [8]. The main purpose of ANOVA is the determination of the highest influence that each design parameters presents [9]. 

Recently, artificial intelligence techniques have been acknowledged as effective and alternative ways for modeling many engineering or other systems precisely [10]. One of these techniques is fuzzy logic, which works on mathematical theory combining multivalued logic and probability theory to overcome complex problems [11]. Fuzzy logic provides extra intelligence and practical means to problem-solving with powerful reasoning capabilities bounded by a minimum number of rules [12].

Multi-spindle drilling using a poly-drill head is used to produce a large number of holes simultaneously, aiming to increase the productivity of the machining process and reduce operation time, hence reducing the overall manufacturing costs [13,14].

Machining parameters play a significant role in any drilling operation where optimal process parameters help in achieving high-quality holes [15]. Several researchers have focused on different methods for the optimization of machining processes, where some of the recent studies are summarized in Table 1.

An overview of the recently cited papers in Table 1 shows the importance of the optimization and prediction of process parameters using statistical design tools and the fuzzy logic approach. Table 1 also shows that optimization processes are commonly used in machining studies to employ the correct analyses, and effectively present that results were statistically analyzed using either one-shot single drilling or any other machining process. Therefore, in this work, the Taguchi method for the optimization of process parameters in multi-hole drilling using a poly-drill head is employed and is compared against one-shot drilling to evaluate its impact on two-hole quality metric: SR and HS. In addition, ANOVA and regression analysis are used to check the model accuracy and the impact of drilling parameters, respectively on the hole quality. Furthermore, the fuzzy logic approach is implemented to measure the values of SR and HS, which are then compared with the experimental results. Finally, validation tests are carried out at the optimal levels of process parameters to verify Taguchi’s method and the simulated values of fuzzy logic.

## 2. Materials and Methods 

**Figure 1:**
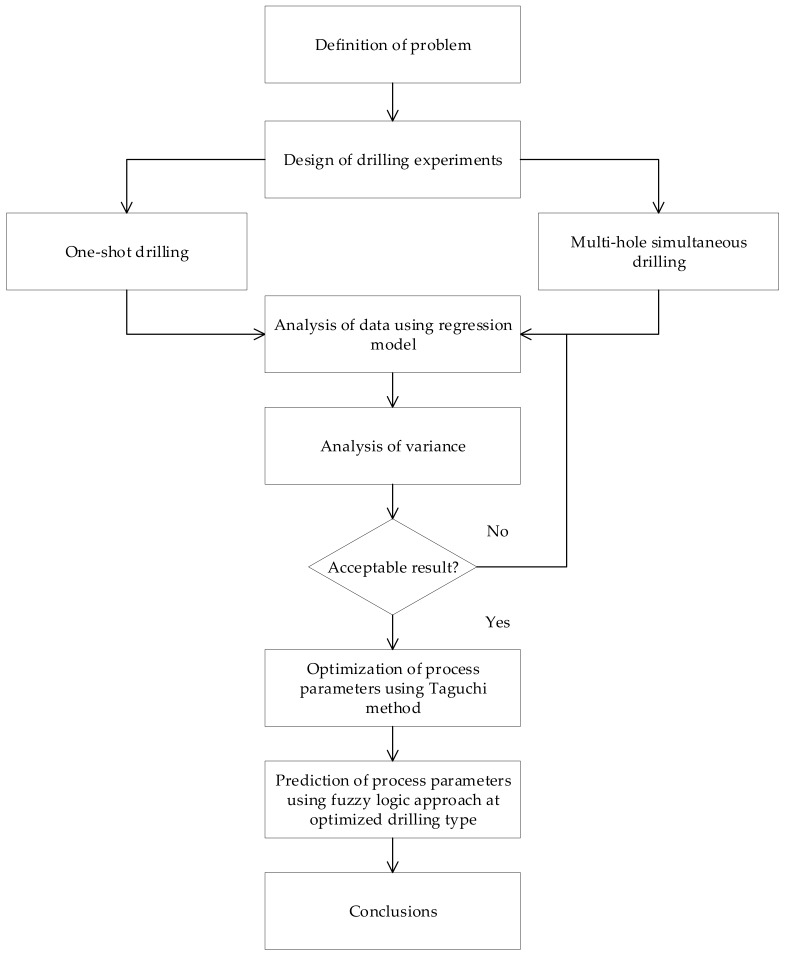
Layout for experimental procedure.

Aluminium alloys have a superior machinability index and are extensively used in various industries such as the aircraft, aerospace, marine, and automotive industries [23]. Therefore, an Aluminium 5083 (Al5083) plate with a thickness of 10 mm and a size of 150 mm × 200 mm was used in this work. The tool material used was 6 mm uncoated high-speed steel (HSS) twist drills with a point angle of 118° and a helix angle of 30° for both one-shot drilling and multi-hole drilling. A new tool in each experiment was used to confirm the initial conditions of each drilling trials. Figure 1 shows different steps of the experimental procedure and verification approach. 

**Figure 2:**
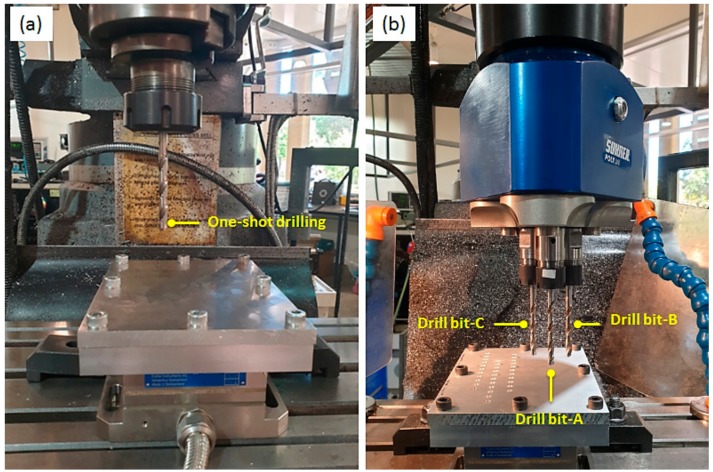
Setup of (**a**) one-shot drilling and (**b**) multi-spindle simultaneous drilling using a poly-drill head.

The drilling experiments were carried under dry drilling conditions using a vertical turret milling machine. Figure 2 shows one-shot drilling and multi-spindle drilling. A SUNHER poly-drill head type MH 30/13 was used for drilling multi-holes simultaneously. 

The surface roughness (SR) of each hole was measured using a roughness tester TR200 (PCWI- precision instrumentation, Australia) equipped with a diamond stylus having a 90° cone angle and a 5 µm tip radius. The ROMER arm which is a balanced measuring instrument integrated with a portable coordinate measuring machine was used to measure the hole size (HS). 

In this study, three drilling parameters including the cutting speed (CS), feed rate (FR), and drilling type (DT) were selected as control factors. A design of experiments (DoE) was employed to determine the relationship between controlled factors (input parameters) affecting the drilling process and the measured output parameters. The information obtained from DoE is needed to manage the drilling process inputs in order to optimize the measured outputs. Full factorial DoE was employed in the current study, which is practical when fewer than five factors are being investigated. The CS and FR were designed to have three levels, and DT has two levels, as given in Table 2. The drilling experiments were conducted according to L_18_ mixed orthogonal array including 18 runs corresponding to several tests for the Taguchi optimization method. The experimental layout with control factors is given in Table 3.

In Taguchi analysis, experimental values are then transformed into a signal-to-noise (S/N) ratio [8] where the term signal refers to the desired values (mean) and noise represent the undesired values (standard deviation) for the output characteristics [2]. In the analysis of the S/N ratio, the quality characteristics are [5] as follows.

Higher the better:

(1) $$S/N\;\mathrm{ratio}\;\left(\eta \right)=\;-10\log_{10}\frac{1}{n}\overset{n}{\underset{i=1}{\sum }}\frac{1}{y_{i}^{2}}$$

Smaller the better:

(2) $$S/N\;\mathrm{ratio}\;\left(\eta \right)=\;-10\log_{10}\frac{1}{n}\overset{n}{\underset{i=1}{\sum }}y_{i}^{2}$$

where y_i_ is the observed response value, and n defines the number of replications.

Nominal the best:

(3) $$S/N\;\mathrm{ratio}\;\left(\eta \right)=\;-10\log_{10}\frac{{µ}^{2}}{\sigma^{2}}$$

where µ = mean and σ = variance.

In the current work, the smaller the better is used to analyze and give the optimal values for the output characteristics i.e., SR and HS. Therefore, optimization of the process parameters was evaluated using the Taguchi method. Furthermore, regression analysis was carried out to correlate between the process parameters and the responses to check the closeness of the developed model with respect to experimental values of SR and HS. Moreover, ANOVA was used to determine the percentage contribution of process parameters on the output response. Finally, fuzzy logic based on Mamdani’s fuzzy inference method was used to simulate the experimental values of the optimal drilling type.

## 3. Results and Discussion

### 3.1. Regression Analysis

In machining processes, regression analysis used to analyze and formulate the correlation between the process parameters and the responses [24]. In this study, the process parameters are the cutting speed (CS), feed rate (FR), and drilling type (DT), where the responses are the surface roughness (SR) and hole size (HS). Therefore, a linear regression model is used to check if the data of SR and HS signify a fitness characteristic. The regression model obtained in this work is given below:

SR = β_0_ + β_1_(DT) + β_2_ (CS) + β_3_ (FR) + ε

SR = 2.728 − 0.401 DT + 0.675 CS + 0.541 FR + ε

R^2^ = 83.89%

HS = β_0_ + β_1_(DT) + β_2_ (CS) + β_3_ (FR) + ε

HS = 6.01961 − 0.00167 DT + 0.007083 CS + 0.010593 FR + ε

**Figure 3:**
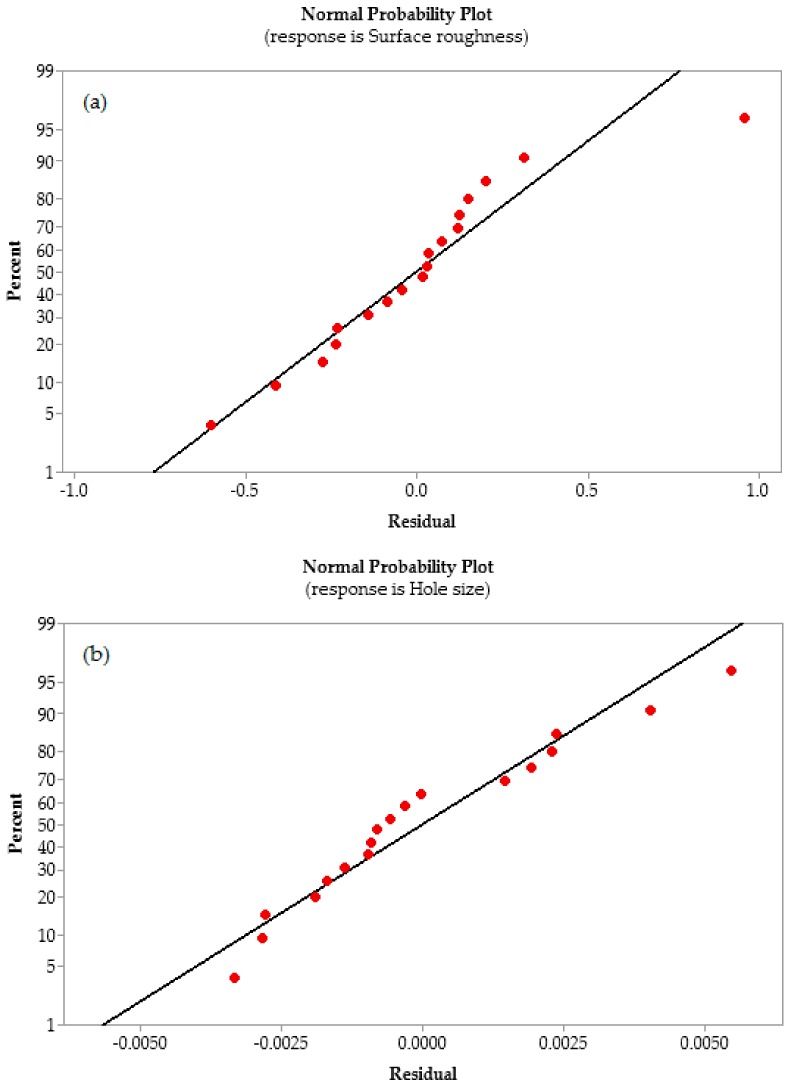
Normal probability plots of residuals for (**a**) surface roughness and (**b**) hole size.

R^2^ = 95.09%

where β_0_ is the constant value when the predictor variables i.e., DT, CS, and FR are zero, β_1_ + β_2_ + β_3_ + β_4_ +…. + βn are the coefficients or estimates of the process parameters, and ε is the error. R^2^ indicates the effectiveness of the developed model. The value equal to 1 shows that the model is 100% effective [25]. Therefore, the larger values of R^2^ are always desirable [26]. In the current study, the values of R^2^ are more than 80%, which indicates that the models are effective in predicting the responses with respect to the machining variables. Figure 3 shows the normal probability plots of the residuals for a predicted response for SR and HS, respectively. Figure 3 also shows that the proposed models are satisfactory as all the residuals follow almost the same pattern of the straight line, which has also been reported by Davidson et al. [27] and Vankanti and Ganta [8]. Therefore, for the selection of process parameters in drilling aluminum, this work will be useful for reducing SR and the deviation of HS from its nominal value.

### 3.2. Optimization of Drilling Conditions Using Taguchi Analysis

As mentioned above, the optimization of drilling parameters in this work is done using Taguchi analysis for better hole quality. Therefore, after a set of experiments given by orthogonal array used in data analysis, the experimental results are then transformed into a S/N ratio, and the smaller the better is used to determine the quality characteristics, as given in Equation (2) [5]. The orthogonal array with control factors, experimental results, and the respective S/N ratio values are displayed in Table 4. 

One of the major characteristics of any machining process is the improvement of SR. SR shows irregularities in the surface of any workpiece due to machining operations [28]. A machined part with high SR results in excessive wear, fatigue, lower material ability to resist corrosion, and lower product performance [29]. SR might be affected by the drilling parameters, tool geometry, and relative vibration induced by the tool on the workpiece. Therefore, measuring SR is important for determining the quality of holes [30]. 

**Figure 4:**
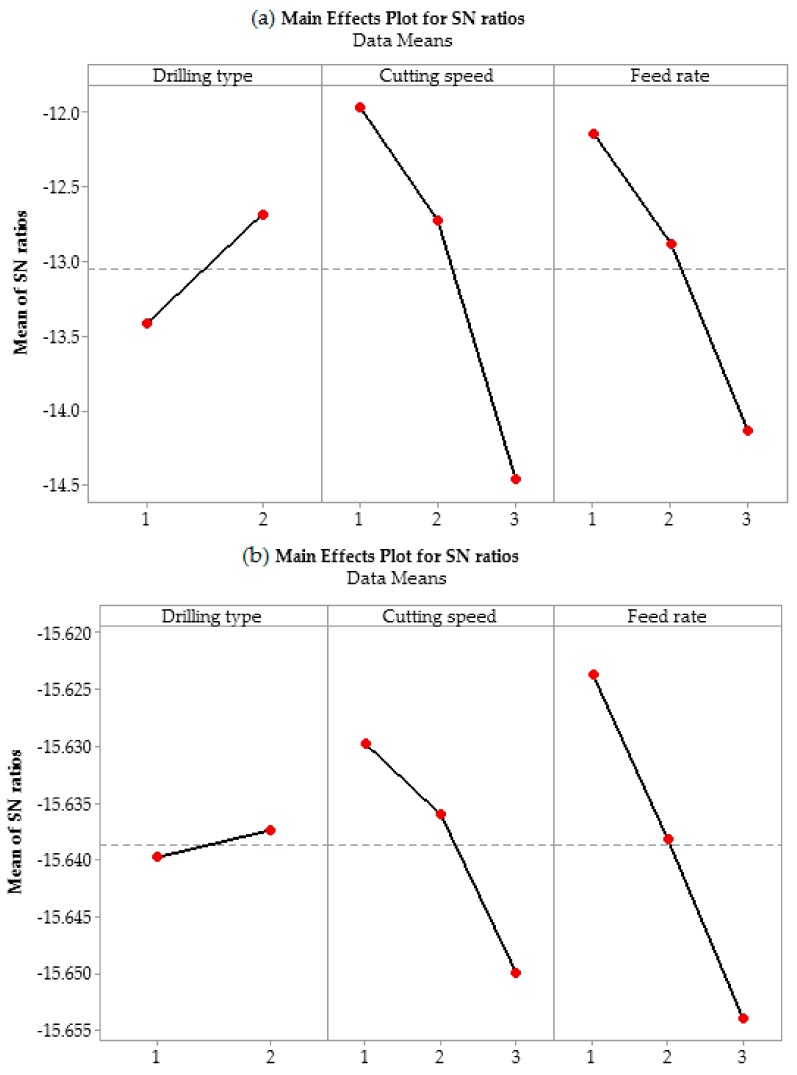
The effect of drilling parameters on (**a**) surface roughness and (**b**) hole size.

Table 5 and 6 show the average response values and S/N ratio values of SR and HS, respectively. Further, the S/N ratio response graph for the SR and HS are shown in Figure 4. According to Yang and Tarng [7], an S/N ratio with higher values leads to better performance, irrespective of the category of the quality characteristic. Therefore, the level of a factor with the highest S/N ratio is the optimum level. As evident in Table 5, the optimal SR is obtained at DT level 2, CS level 1, and FR level 1. This shows that at lower drilling parameters, multi-spindle drilling using a poly-drill head gives better SR. Lower CS and FR are also recommended by Giasin et al. [31], as higher CS might cause more vibration and chatter, which reduces the surface quality. Furthermore, a higher FR increases the chip thickness, which might also result in a higher SR of holes [32].

As mentioned earlier, all the drilling tools used in this study have a 6 mm diameter. Therefore, it is important to check if there is any deviation from the nominal size. Kurt et al. [29] reported in their studies that HS is also influenced by drilling parameters, where the hole diameter may increase from the nominal size. The reason for this was attributed to the fact that at high drilling parameters, there are higher chances of vibration due to the dynamic behaviour of the tool. Furthermore, Roukema and Altintas [33] have shown that there is an increased possibility of temperature in the tool and workpiece at high drilling parameters, which could adversely affect the drilled-holes and hinder chip evacuation and thus affect the HS [29]. Therefore, the selection of optimal drilling parameters is necessary for the measurement of HS. From Table 6 and Figure 4, the optimum conditions for HS can be selected. The analysis of S/N ratio shows that the poly-drill head also performs better than one-shot drilling, as the best combination of parameters can be obtained at DT level 2, CS level 1, and FR level 1.

Lower FR facilitated the slower insertion of the tool, which allowed a stable and jerk-free performance as the cutting edges of the tool removed the material with smaller chip thickness; consequently, the HS shows less dimensional error [34]. Furthermore, at higher CS, chatter and drilling temperature appeared to be more, which might affect the size of the hole [29]. Therefore, it can be concluded that lower FR is recommended for lower SR and better dimensional accuracy. However, while a lower FR affects productivity, a poly-drill head gave three holes at the same drilling time, which would help in acquiring good productivity even when using a lower FR.

### 3.3. Prediction of Surface Roughness and Hole Size at Optimal Levels

The values of SR and HS at their optimal level of process parameters can be predicted as follows.

The predicted mean at the optimal process parameters:

SR = (Level)_2_ + (Level)_1_ + (Level)_1_ – 2 (Y) = 4.358 + 3.991 + 4.082 − 2 (4.559) = 3.313

HS = (Level)_2_ + (Level)_1_ + (Level)_1_ – 2 (Y) = 6.052 + 6.046 + 6.042 − 2 (6.052) = 6.035

where (Level)_2_, (Level)_1_, and (Level)_1_ are the optimal levels of SR and HS obtained from Table 5 and Table 6, respectively. Y is the overall mean of experimental values calculated from Table 4. Similarly, the corresponding S/N ratio can be calculated to check whether the SR and HS values are acceptable.

The predicted S/N ratio (maximum):

SR = (S/N)_2_ + (S/N)_1_ + (S/N)_1_ – 2 (S/N)_m_ = (−12.685) + (−11.969) + (−12.142) − 2 (−13.054) = −10.688

HS = (S/N)_2_ + (S/N)_1_ + (S/N)_1_ – 2 (S/N)_m_ = (−15.637) + (−15.630) + (−15.624) − 2 (−15.639) = −15.614

where (S/N)_2_, (S/N)_1_, and (S/N)_1_, are the maximum values of S/N ratios of SR and HS at the optimal levels obtained from Table 5 and Table 6, respectively. (S/N)_m_ is the overall mean of S/N ratios calculated from Table 4.

### 3.4. Analysis of Variance

ANOVA has been applied in this study to measure the importance of each of the process parameters on SR and HS. Table 7 and Table 8 show the ANOVA results for the SR and HS, respectively. The percent contribution in ANOVA is used to describe how much influence each process parameters has on the output responses. The P-value can confirm the effect of process parameters on responses and shows that values less than 0.05 have no effect [35]. Another statistical tool is F-value, which is used to check the design parameters with a significant impact on the quality characteristic [2]. The confidence interval chosen in this study is 95% (α = 0.05). 

Accordingly, Table 7 shows that the statistical influences of CS and FR on SR were 40.16% and 25.02%, respectively. This shows that the CS has more impact on hole roughness than the FR. Similar findings were also reported by Kilickap [23] when drilling aluminium alloys. However, in the case of HS, the influence of FR was more than the CS value given in Table 8. The percentage contribution of FR is 61.31%, which is more than the contribution of CS with a statistical significance of 28.72%. 

### 3.5. Fuzzy Modeling for Surface Roughness and Hole Size

**Figure 5:**
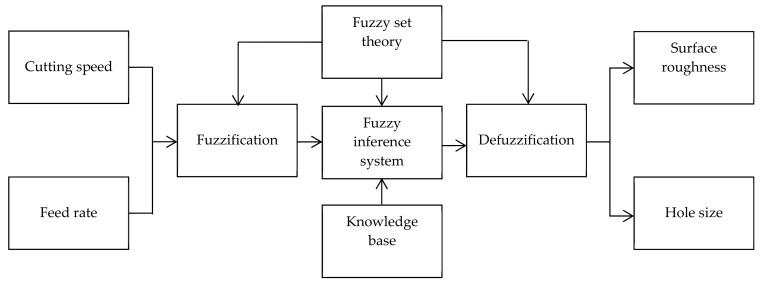
Schematic diagram of the developed algorithm.

From the aforesaid Taguchi analysis, it is apparent the multi-hole drilling using a poly-drill head gives better SR and HS than those of one-shot drilling. Therefore, the experimental data of the poly-drill head are used for the fuzzy-based algorithm to predict SR and HS at different process parameters. In this regard, a conceptual framework based on the fuzzy logic algorithm is designed in MATLAB® (R2018b) and is illustrated in Figure 5.

The fuzzy modeling is comprised of three steps that include the fuzzification process, rule framing process, and defuzzification. In the fuzzification process, with the help of the membership definition, each factor is treated as a well-defined numeric value. In the second stage, rules are designed by the combination of parameters using logical operators. Finally, defuzzification is done in which all membership degrees are made into a quantifiable value by combining the outputs of the framed rules [36]. 

**Figure 6:**
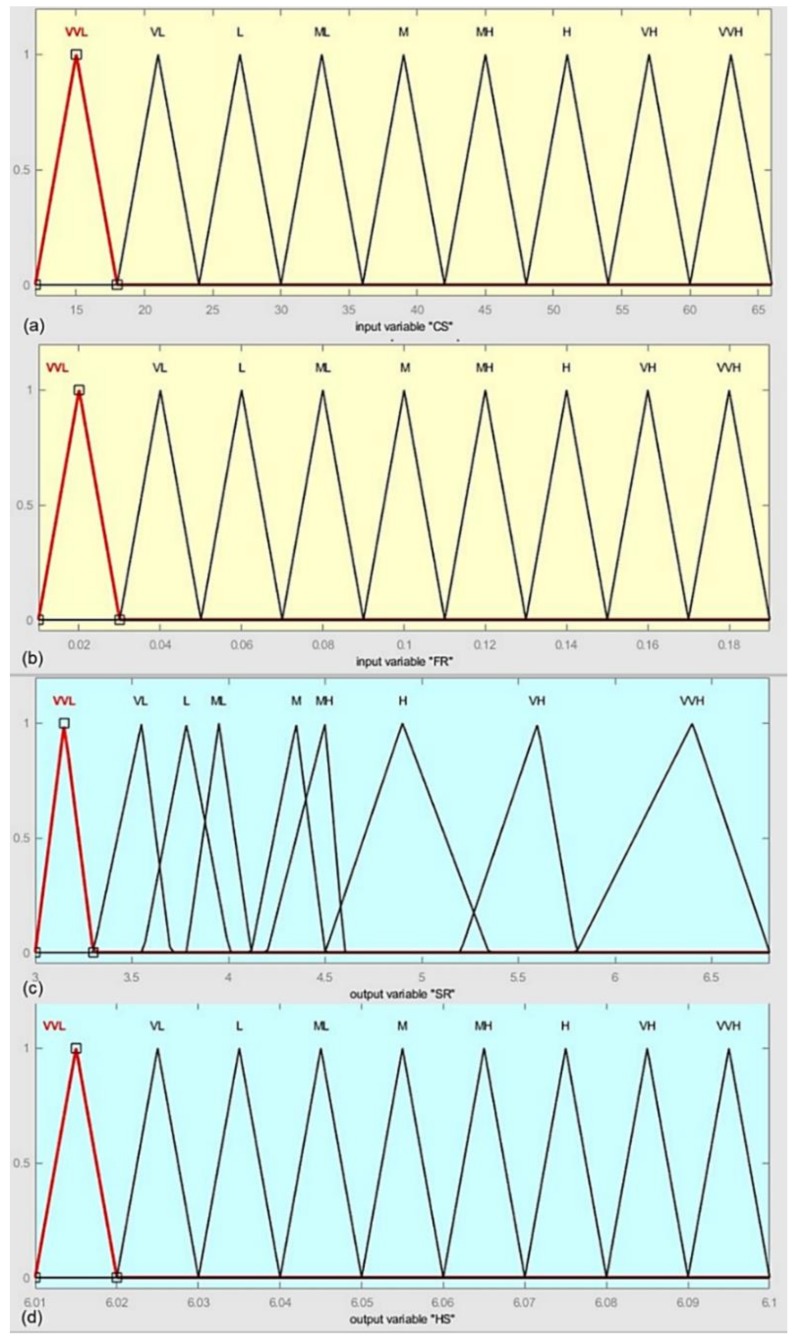
Membership functions: (**a**) input membership function for cutting speed; (**b**) input membership function for feed rate; (**c**) output membership function for surface roughness; (**d**) output membership function for hole size.

CS and FR are taken as input in the fuzzy inference system where the SR and HS are considered as output. A fuzzy-based rule is defined that predicts SR and HS in the fuzzy domain, and the fuzzy interference engine is considered as “Mamdani”. Mamdani shows relatively better results [37]. Therefore, in modeling the algorithm, the outputs of the system are calculated based on the centroid method, and Mamdani implication is used for defuzzification. A triangular membership function (MF) is considered in this study. In the developed algorithm, the input and output variables are fuzzified into nine fuzzy sets: very very low (VVL), very low (VL), low (L), moderately low (ML), medium (M), moderately high (MH), high (H), very high (VH), and very very high (VVH). The associated MFs as input variables and output variables are shown in Figure 6, and the detailed parameters of fuzzy inference system with the triangular MFs are given in Table 9.

The developed algorithm was tested on the experimental data of the multi-hole drilling tests for SR and HS. It was found that the algorithm was able to detect all the values at the desired process parameters. The values in the fuzzy set from the developed algorithm behave similarly to the experimental values in which an increase in the drilling parameters increases SR and HS. Table 10 shows that both the experimental data and fuzzy results are consistent with each other and behave similarly for SR and HS. Similarly, a good match is observed in the experimental and modeled values, showing that at high drilling parameters, there is more deviation of the HS from nominal size. In both cases, the percentage error occurs within the range of 10% for the validation of experimental results. The predicted values are in good agreement with experimental values, which reflects that the obtained fuzzy models present a feasible and effective way for the prediction of SR and HS in a drilling process using a poly-drill head for multi-hole simultaneous drilling.

### 3.6. Validations of Results

**Figure 7:**
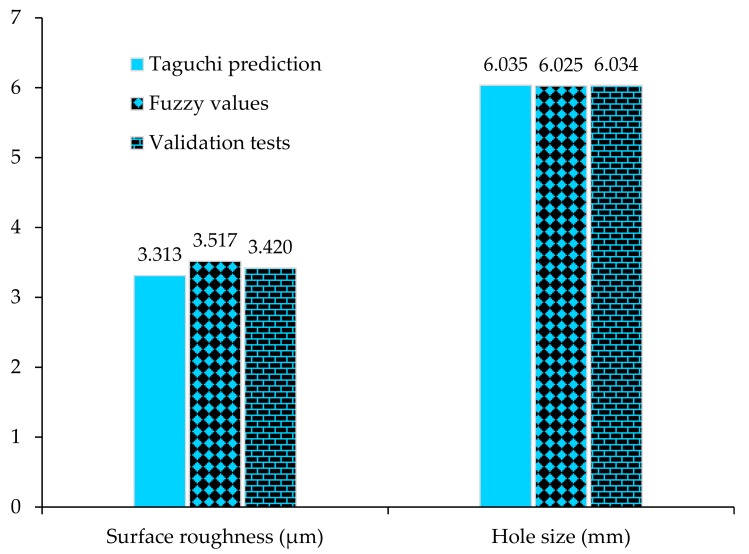
Validation of experimental results for surface roughness and hole size.

The validation tests are an essential step for the verification of the results obtained from Taguchi’s design approach [38]. Therefore, validation experiments at the optimized process parameters are conducted for SR and HS using a poly-drill head. The average of the validation results is compared with the predicted average based on the Taguchi optimal process parameters and fuzzy logic values. Figure 7 shows that there is a small difference among all the values, and the response optimization predicts the optimum conditions comparatively well. 

## 4. Conclusions

Optimal machining parameters are essential for high quality drilling of holes, which leads to efficient and effective production. In this work, drilling experiments were performed using one-shot drilling and multi-hole simultaneous drilling with a poly-drill head. The Taguchi method was used for the optimization of process parameters for better hole quality in terms of surface roughness and hole size. Then, regression analysis and ANOVA were applied to confirm the accuracy of the model and the significance of process parameters. Moreover, a fuzzy logic technique was employed for the prediction of surface roughness and hole size in multi-hole drilling using a poly-drill head. The following conclusions are drawn from this work:

- Cutting speed and feed rate in the drilling of Al5083 have a significant impact on both types of drilling process i.e., one-shot drilling and multi-hole drilling. A higher cutting speed generates more heat, which increases the temperature at the hole boundaries, where the chips can easily be clogged over the flutes of the drills. Furthermore, it is speculated that more vibration is likely to be present due to the rotary motion of the tool. In addition, the size of the chips increases with an increase in the feed rate. Therefore, a higher cutting speed and feed rate can affect both the surface roughness and hole size.
- The ANOVA results revealed that for both drilling types, the cutting speed and feed rate are influential on the surface roughness and hole size, respectively. The percent influence of cutting speed on the surface roughness is 40.16%, whereas the feed rate has a 25.02% contribution. In the case of hole size, the deviation from the nominal drill size is more affected by the feed rate, which has a 61.31% contribution, and the significance of the cutting speed is 28.72%.
- The Taguchi method was shown to successfully analyze the optimal combination of process parameters for surface roughness and hole size, as achieved in multi-hole drilling with a lower cutting speed and feed rate. Therefore, in comparison to one-shot drilling, a poly-drill head can produce better hole quality and is capable of forming multi-holes simultaneously, which would help in acquiring good productivity even at the lower levels of drilling parameters.
- The fuzzy modeling successfully investigated the surface roughness and hole size in multi-hole drilling. The predicted data is closely correlated to the experimental results with a very small percentage error. Thus, the developed fuzzy model is reliable for the prediction of hole quality at different levels of process parameters, where notable savings in time and cost could be obtained.
- In regression analysis, the values of R^2^ are more than 80%, which shows that the responses with respect to the machining variables could be easily predicted. The validation experiment reveals that the developed model can be used for predicting better hole quality using a poly-drill head for multi-hole drilling. The average error between each comparison was found to be small and hence, the optimization by the Taguchi method and prediction of values for surface roughness and hole size using fuzzy logic are both acceptable.

## Figures and Tables

**Table 1:** Summary of different optimization techniques used for predicting process parameters in previous machining studies.

Process	Material	Considered Process Parameters	Objectives	Optimization/Prediction Technique	Ref
**OSD**	AISI 4140 steel	DG, CS, and FR	CYL, PER, CON	Taguchi, ANOVA	[16]
**OSD**	CFRP	SS, FR, and D	TF, T, DL	ANOVA analysis, Fuzzy logic	[17]
**OSD**	Hybrid polymer composites	SS, FR, and D	TF, T, DL	Gray relational analysis, regression, fuzzy logic, and artificial neural network models	[10]
**Milling**	hardened Steel (steel 1.2738)	CS, FR, radial depth, and axial depth	SR	Taguchi optimization technique, ANOVA,	[9]
**OSD**	Al6063/Al2O3/Gr hybrid composite	SS, FR, and wt % of alumina	SR	Taguchi method	[18]
**OSD**	CFRP	SS, FR, and D	TF, T, DL	Taguchi method, Principal component analysis, Fuzzy inference system	[19]
**OSD**	GFRP	SS, FR, point angle, and chisel edge width	TF, T, SR, C	Taguchi, ANOVA	[8]
**OSD**	Al 7075	Tool, FR, and CS	TEMP	Taguchi method, ANOVA	[20]
**End milling**	GRFP	SS, FR, and d	TL	Fuzzy logic	[21]
**Turning**	CFRP	CS, feed, and d	SR	Fuzzy rule-based modeling	[22]
**OSD**	Al-7075	CS, FR, and point angle,	H, SR	Taguchi method and response surface methodology	[23]
**OSD**	GFRP	SS, FR, and D	SR	Fuzzy logic and ANOVA	[11]
**OSD**	Al 2024	Drilling depth, FR, CS, and drilling tool	Diametral error, SR	Regression model, Taguchi optimization method, ANOVA	[2]

Cutting speed: CS, Drill geometry: DG, Drill diameter: D, surface roughness: SR, Spindle speed: SS, Feed rate: FR, Depth of cut: d, Thrust force: TF, Torque: T, Delamination: DL, Circularity: C, Burr height: H, Perpendicularity: PER, Temperature: TEMP, Tool Life: TL, Concentricity: CON, Cylindricity: CYL, One-shot drilling: OSD.

**Table 2:** Process parameters and their levels.

Levels	Process Parameters		
	Drilling Type	Cutting Speed	Feed Rate
1	One-shot drilling	19	0.04
2	Multi-spindle drilling	38	0.08
3	–	57	0.14

**Table 3:** Experimental layout with control factors.

Experiment No.	Control Factors		
	Drilling Type	Cutting Speed	Feed Rate
1	One-shot drilling	19	0.04
2		19	0.08
3		19	0.14
4		38	0.04
5		38	0.08
6		38	0.14
7		57	0.04
8		57	0.08
9		57	0.14
10	Multi-spindle drilling	19	0.04
11		19	0.08
12		19	0.14
13		38	0.04
14		38	0.08
15		38	0.14
16		57	0.04
17		57	0.08
18		57	0.14

**Table 4:** Experimental results according to orthogonal array with signal-to-noise (S/N) ratio.

Trial No.	Orthogonal Array with Control Factors			Experimental Results		S/N Ratio	
	Drilling Type	Cutting Speed	Feed Rate	Surface Roughness	Hole Size	Surface Roughness	Hole Size
1	1	1	1	3.561	6.040	−11.030	−15.620
2	1	1	2	4.209	6.048	−12.483	−15.632
3	1	1	3	4.657	6.056	−13.362	−15.644
4	1	2	1	4.422	6.043	−12.912	−15.625
5	1	2	2	4.522	6.052	−13.107	−15.638
6	1	2	3	4.699	6.062	−13.441	−15.652
7	1	3	1	4.808	6.047	−13.639	−15.631
8	1	3	2	5.022	6.059	−14.017	−15.648
9	1	3	3	6.933	6.073	−16.818	−15.669
10	2	1	1	3.457	6.036	−10.775	−15.615
11	2	1	2	3.718	6.044	−11.405	−15.627
12	2	1	3	4.344	6.054	−12.757	−15.641
13	2	2	1	3.676	6.039	−11.307	−15.620
14	2	2	2	4.126	6.049	−12.311	−15.633
15	2	2	3	4.626	6.059	−13.304	−15.648
16	2	3	1	4.566	6.048	−13.191	−15.632
17	2	3	2	4.990	6.061	−13.961	−15.651
18	2	3	3	5.724	6.075	−15.154	−15.671

Cutting speed (m/min), Feed rate (mm/rev), Surface roughness (μm), Hole size (mm).

**Table 5:** Average response values and S/N ratios for surface roughness.

Level	Surface Roughness					
	Average Response Values			S/N Ratio Response Values		
	Drilling Type	Cutting Speed	Feed Rate	Drilling Type	Cutting Speed	Feed Rate
**1**	4.759	**3.991 ^a^**	**4.082 ^a^**	−13.423	**−11.969 ^a^**	**−12.142 ^a^**
2	**4.358 ^a^**	4.345	4.431	**−12.685 ^a^**	−12.730	−12.881
3	–	5.340	5.164	–	−14.463	−14.139

^a^ Optimum value.

**Table 6:** Average response values and S/N ratios for hole size.

Level	Hole size					
	Average Response Values			S/N Ratio Response Values		
	Drilling Type	Cutting Speed	Feed Rate	Drilling Type	Cutting Speed	Feed Rate
1	6.053	**6.046 ^a^**	**6.042 ^a^**	−15.640	**−15.630 ^a^**	**−15.624 ^a^**
2	**6.052 ^a^**	6.051	6.052	**−15.637 ^a^**	−15.636	−15.638
3	–	6.060	6.063	–	−15.650	−15.654

^a^ Optimum value.

**Table 7:** ANOVA for surface roughness.

Source	Degrees of Freedom	Sequential Sum of Squares	Contribution	Adjusted Sum of Squares	Adjusted Mean Square	F-Value	P-Value
Model	19	36.0261	82.08%	36.0261	1.89611	8.2	0
CS	2	17.6251	40.16%	17.6251	8.81254	38.09	0
FR	2	10.9826	25.02%	10.9826	5.49129	23.74	0
DT	1	2.1673	4.94%	2.1673	2.16731	9.37	0.004
2-Way Interactions	8	2.4065	5.48%	2.4065	0.30081	1.3	0.276
CS x FR	4	2.2008	5.01%	2.2008	0.55019	2.38	0.071
CS x DT	2	0.0825	0.19%	0.0825	0.04126	0.18	0.837
FR x DT	2	0.1232	0.28%	0.1232	0.06159	0.27	0.768
3-Way Interactions	4	1.5096	3.44%	1.5096	0.3774	1.63	0.189
CS x FR x DT	4	1.5096	3.44%	1.5096	0.3774	1.63	0.189
Error	34	7.8658	17.92%	7.8658	0.23135	–	–
Total	53	43.8919	100.00%	–	–	–	–

**Table 8:** ANOVA for hole size.

Source	Degrees of Freedom	Sequential Sum of Squares	Contribution	Adjusted Sum of Squares	Adjusted Mean Square	F-Value	P-Value
Model	19	0.00622	94.34%	0.00622	0.000327	29.84	0
CS	2	0.001893	28.72%	0.001893	0.000947	86.31	0
FR	2	0.004042	61.31%	0.004042	0.002021	184.24	0
DT	1	0.000037	0.57%	0.000037	0.000037	3.42	0.073
2-Way Interactions	8	0.000211	3.21%	0.000211	0.000026	2.41	0.035
CS × FR	4	0.000148	2.24%	0.000148	0.000037	3.37	0.02
CS × DT	2	0.000061	0.93%	0.000061	0.00003	2.78	0.076
FR × DT	2	0.000002	0.04%	0.000002	0.000001	0.11	0.896
3-Way Interactions	4	0.000003	0.04%	0.000003	0.000001	0.06	0.992
CS × FR × DT	4	0.000003	0.04%	0.000003	0.000001	0.06	0.992
Error	34	0.000373	5.66%	0.000373	0.000011	–	–
Total	53	0.006593	100%	–	-	–	–

**Table 9:** Parameter of fuzzy inference system with membership functions and variables. VVL: very very low, VL: very low, L: low, ML: moderately low, M: medium, MH: moderately high, H: high, VH: very high, VVH: very very high.

Membership Function Type	Variable	Fuzzy Input				Fuzzy Output			
		Cutting Speed		Feed Rate		Surface Roughness		Hole Size	
		Parameter	Range	Parameter	Range	Parameter	Range	Parameter	Range
**Triangular**	VVL	(12 15 18)	(12 66)	(0.01 0.02 0.03)	(0.01 0.19)	(3 3.15 3.3)	(3 6.8)	(6.01 6.015 6.02)	(6.01 6.1)
	VL	(18 21 24)	(12 66)	(0.03 0.04 0.05)	(0.01 0.19)	(3.3 3.55 3.7)	(3 6.8)	(6.02 6.025 6.03)	(6.01 6.1)
	L	(24 27 30)	(12 66)	(0.05 0.06 0.07)	(0.01 0.19)	(3.559 3.783 4.005)	(3 6.8)	(6.03 6.035 6.04)	(6.01 6.1)
	ML	(30 33 36]	(12 66)	(0.07 0.08 0.09)	(0.01 0.19)	(3.783 3.95 4.117)	(3 6.8)	(6.04 6.045 6.05)	(6.01 6.1)
	M	(36 39 42)	(12 66)	(0.09 0.1 0.11)	(0.01 0.19]	(4.117 4.35 4.5)	(3 6.8]	(6.05 6.055 6.06)	(6.01 6.1)
	MH	(42 45 48)	(12 66)	(0.11 0.12 0.13)	(0.01 0.19)	(4.2 4.5 4.6)	(3 6.8)	(6.06 6.065 6.07)	(6.01 6.1)
	H	(48 51 54)	(12 66)	(0.13 0.14 0.15)	(0.01 0.19)	(4.5 4.9 5.347)	(3 6.8)	(6.07 6.075 6.08)	(6.01 6.1)
	VH	(54 57 60)	(12 66)	(0.15 0.16 0.17)	(0.01 0.19)	(5.2 5.6 5.8)	(3 6.8)	(6.08 6.085 6.09)	(6.01 6.1)
	VVH	(60 63 66)	(12 66)	(0.17 0.18 0.19)	(0.01 0.19)	(5.8 6.4 6.8)	(3 6.8)	(6.09 6.095 6.1)	(6.01 6.1)

**Table 10:** Experimental values and fuzzy results with percentage errors.

Hole Number	Cutting Speed	Feed Rate	Surface Roughness			Hole Size		
			Experimental Values	Fuzzy Results	% Error	Experimental Values	Fuzzy Results	% Error
1	19	0.04	3.457	3.517	−1.736	6.036	6.025	0.182
2	19	0.08	3.718	3.774	−1.506	6.044	6.038	0.101
3	19	0.14	4.344	4.622	−6.400	6.055	6.057	−0.035
4	38	0.04	3.676	3.888	−5.767	6.039	6.039	−0.003
5	38	0.08	4.126	4.136	−0.242	6.048	6.050	−0.028
6	38	0.14	4.626	4.745	−2.572	6.060	6.066	−0.092
7	57	0.04	4.566	4.728	−3.548	6.048	6.055	−0.116
8	57	0.08	4.990	4.962	0.561	6.061	6.065	−0.064
9	57	0.14	5.724	5.170	9.679	6.075	6.080	−0.082
